# Full-Length Transcriptome Sequencing and RNA-Seq Analysis Offer Insights into Terpenoid Biosynthesis in *Blumea balsamifera* (L.) DC.

**DOI:** 10.3390/genes15030285

**Published:** 2024-02-24

**Authors:** Zhigang Ju, Lin Liang, Yaqiang Zheng, Hongxi Shi, Wenxuan Zhao, Wei Sun, Yuxin Pang

**Affiliations:** 1 Pharmacy College, Guizhou University of Traditional Chinese Medicine, Guiyang 550025, China; juzhigang088@gzy.edu.cn (Z.J.); liangl187@163.com (L.L.); zhengyaqiang131@gzy.edu.cn (Y.Z.); shihongxi055@gzy.edu.cn (H.S.); zhaowenxuan0602@163.com (W.Z.); 2 Key Laboratory of State Forestry Administration on Biodiversity Conservation in Karst Mountain Area of Southwest of China, School of Life Science, Guizhou Normal University, Guiyang 550025, China; 3 Yunfu Branch, Guangdong Laboratory for Lingnan Modern Agriculture, Yunfu 527300, China

**Keywords:** *Blumea balsamifera*, essential oil, SMART, terpene synthase, *L*-borneol

## Abstract

*Blumea balsamifera* (L.) DC., an important economic and medicinal herb, has a long history of being used as a traditional Chinese medicine. Its leaves have always been used as a raw material for the extraction of essential oils, comprising large amounts of terpenoids, which have good therapeutic effects on many diseases, such as eczema, bacterial infection, and hypertension. However, the genetic basis of terpenoid biosynthesis in this plant is virtually unknown on account of the lack of genomic data. Here, a combination of next-generation sequencing (NGS) and full-length transcriptome sequencing was applied to identify genes involved in terpenoid biosynthesis at five developmental stages. Then, the main components of essential oils in *B. balsamifera* were identified using GC–MS. Overall, 16 monoterpenoids and 20 sesquiterpenoids were identified and 333,860 CCS reads were generated, yielding 65,045 non-redundant transcripts. Among these highly accurate transcripts, 59,958 (92.18%) transcripts were successfully annotated using NR, eggNOG, Swissprot, KEGG, KOG, COG, Pfam, and GO databases. Finally, a total of 56 differently expressed genes (DEGs) involved in terpenoid biosynthesis were identified, including 38 terpenoid backbone genes and 18 TPSs, which provide a significant amount of genetic information for *B. balsamifera*. These results build a basis for resource protection, molecular breeding, and the metabolic engineering of this plant.

## 1. Introduction

*B. balsamifera* (L.) DC., a perennial Compositae plant, has long been used as an ethnic medicine in southwest China, including in Guizhou, Guangxi, Yunan, and Hainan Provinces [1,2]. *B. balsamifera* located in Luodian County of Guizhou Province has become a geographical indication protection product, and its planting area has reached as much as 30,000 hectares. Since 2010, the Pharmacopoeia of the People’s Republic of China recorded *B. balsamifera* as the only plant source for “Ai Pian”, which can induce resuscitation, reduce a fever, and relive pain. Due to its high quantity of essential oils, *B. Balsamifera* is also named “Ai Na Xiang” and “Da Feng Ai” in Chinese and has become an important economic crop in the local area [3]. Moreover, the essential oils of *B. balsamifera* display new physiological activities, such as anti-tumor [4,5,6], anti-fungal [7,8], radical-scavenging [9], and anti-influenza virus properties [10].

The major active compounds of essential oils in *B. balsamifera* are monoterpenoids and sesquiterpenoids, such as *L*-borneol, linalool, camphor, and pinene. The biosynthesis pathways of terpenoids are well understood in many plants [11,12]. Generally, the mevalonate acid pathway (MVA) and the methylerythritol pathway (MEP) are responsible for the biosynthesis of isopentenyl diphosphate (IPP) and dimethylallyl diphosphate (DMAPP), respectively. IPP and DMAPP can be condensed into geranyl pyrophosphate (GPP), farnesyl pyrophosphate (FPP), geranyl geranyl pyrophosphate (GGPP), and geranyl farnesyl pyrophosphate (GFPP) [13,14,15]. With the catalysis of terpenoid synthases (TPSs), GPP is further transformed into mono-, di-, and tetra-terpenoids, while FPP is transferred into sesquiterpenoids and polyterpenoids [16,17]. 

However, the biosynthesis pathways of the terpenoids in *B. balsamifera* are rarely reported. Importantly, no genomic information for *B. balsamifera* is available. This is unfavorable for the elucidation of the regulatory mechanisms of the growth and development of *B. balsamifera*, especially hub genes regulating the biosynthesis of terpenoids in essential oils, which restricts the development of molecular breeding and the precision cultivation of *B. balsamifera*. The advent of full-length transcriptome sequencing opens a path for the mining of genetic information of many medicinal plants [18,19,20,21,22,23]. In this study, a combination of NGS and full-length transcriptome sequencing was used to obtain a more comprehensive transcriptome. Meanwhile, DETs involved in the biosynthesis of monoterpenoids and sesquiterpenoids were also identified. Moreover, the metabolomics of the leaves of *B. balsamifera* were also detected. This study provides novel insights into terpenoid biosynthesis in *B. balsamifera*.

## 2. Results

### 2.1. Terpenoids of the Essential Oils in B. balsamifera

The essential oils extracted from the leaves of *B. balsamifera* were identified using GC–MS/MS based on the MWGC database. As shown in Table 1, two major types of terpenoids were detected, including 16 monoterpenoids and 20 sesquiterpenoids (Table 1). For monoterpenoids, *L*(-)-borneol showed the highest content followed by (-)-terpinen-4-ol, linalool, (-)-orthodene, and comphene. For sesquiterpenoids, 2-methylene-4,8,8-trimethyl-4-vinylbicyclo [5.2.0] nonane showed the highest content followed by 1R,4R,7R,11R-1,3,4,7-Tetramethyltricyclo[5.3.1.0(4,11)]undec-2-ene, *β*-copaene, (-)-*α*- gurjunene, and (-)-*epi*-bicyclosesquiphellandrene. These results showed that monoterpenoids and sesquiterpenoids are the main components of these essential oils.

### 2.2. Transcriptome Assembly from NGS and SMRT Sequencing of B. balsamifera

To deeply mine the genetic information of *B. balsamifera*, transcriptome sequencing was performed using the PacBio and Illumina platforms simultaneously. For RNA-Seq, 15 mRNA samples from the leaves of *B. balsamifera* at five different developmental stages (each in triplicate) were used to construct libraries for NGS using Illumina HiSeq. After trimming and filtering, 26,306,866 (BBLI), 27,130,069 (BBLII), 22,917,945 (BBLIII), 27,682,295 (BBLIV), and 23,295,190 (BBLV) clean reads were obtained. The average GC percentage and Q30 of all libraries were 44.97% and 95.48%, respectively (Table S1). The integrity of the transcriptome was also assessed and illustrated in Figure S1.

For full-length transcriptome sequencing, 1–6 kb full-length cDNA libraries containing BBLI, BBLII, BBLIII, BBLIV, BBLV, BBR, and BBS samples were constructed and applied for SMRT sequencing using the PacBio platform. A total of 333,860 reads of inserts (ROIs) were generated with an average length of 2244 bp. After circular consensus sequence (CCS) generation and filtering for full-length read classification, 298,671 full-length non-chimeric (FLNC) reads were retained. The FLNC reads were clustered into consensus clusters based on RS IsoSeq. Then, 116,656 high-quality (HQ) and consensus isoforms were merged into a total of 116,639 final consensus sequences (Table 2). Finally, a total of 65,045 non-redundant full-length transcripts from *B. balsamifera* were generated using CD-HIT.

### 2.3. Functional Annotation of Full-Length Transcriptome

**Figure 1:**
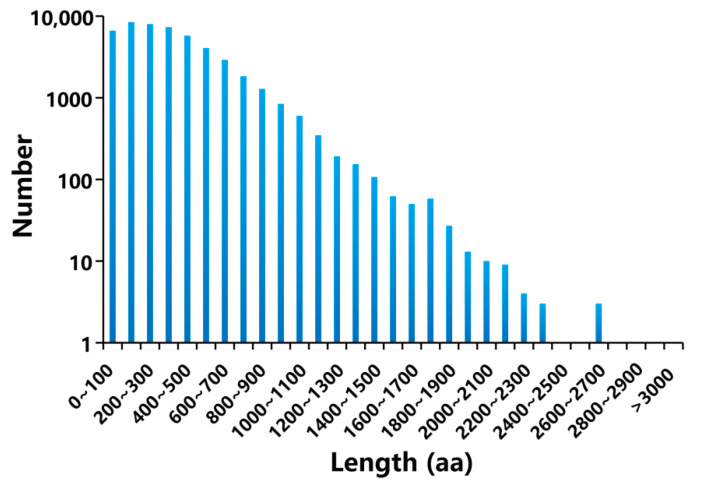
The length distribution of CDSs.

**Figure 2:**
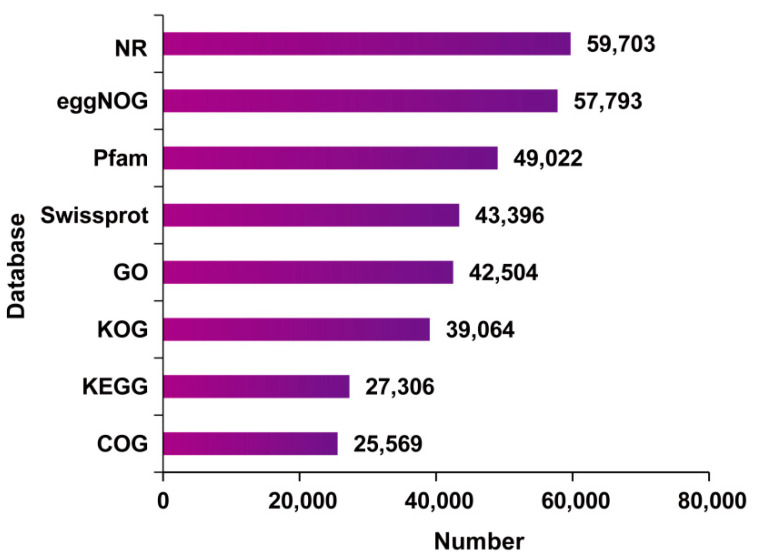
The number of annotated unigenes with various databases.

A total of 65,045 complete ORFs were obtained using TransDecoder. As illustrated in Figure 1, only a minority (29 CDSs) had more than 2100 base pairs (bp), and 86.68% of the CDSs appeared with a length ranging from 100 to 2100 bp. Then, all non-redundant unigenes were blasted against eight biological databases. The results showed that 59,958 unigenes were functionally annotated (Figure 2). Among them, 25,569 (39.41%), 27,306 (41.98%), 39,064 (60.06%), 42,504 (65.35%), 43,396 (66.72%), 49,022 (75.37%), 57,793 (88.85%), and 59,703 (91.79%) unigenes were annotated in COG, KEGG, KOG, GO, Swissprot, Pfam, eggNOG, and Nr, respectively (Table S2).

**Figure 3:**
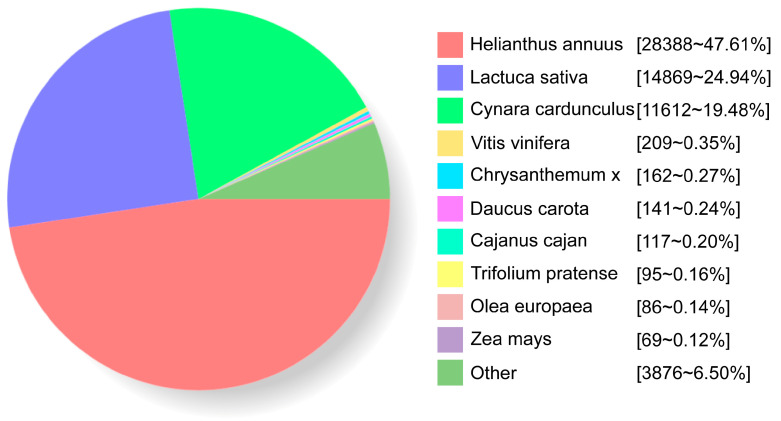
Species homologous to *B. balsamifera*.

By aligning the obtained sequences with the NR database, species homologous to *B. balsamifera* were analyzed (Figure 3). As a result, 47.61%, 24.94%, and 19.48% of the sequences were mapped to the genes of *Helianthus annuus* (Arsteraceae), *Lactuca sativa* (Arsteraceae), and *Cynara cardunculus* (Arsteraceae), respectively.

**Figure 4:**
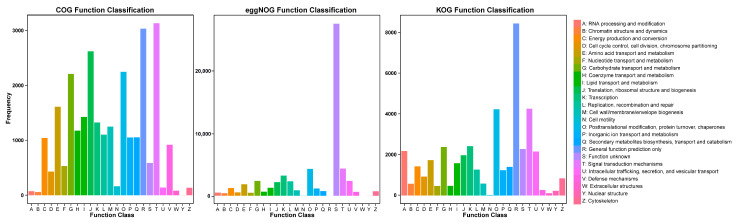
COG, eggNOG, and KOG functional annotation.

Furthermore, 25,569, 39,064, and 57,793 unigenes were assigned using the COG, KOG, and eggNOG databases, respectively, and classified into 25 functional clusters (Figure 4; Table S3). In the COG functional annotation, “signal transduction mechanisms” (3132 unigenes) was the most common annotation followed by “general function prediction” (3032 unigenes) and “translation, ribosomal structure and biogenesis” (2620 unigenes). In the KOG functional annotation, the most common annotation was “general function prediction” (8455 unigenes) followed by “signal transduction mechanisms” (4268 unigenes) and “posttranslational modification, protein turnover, chaperones” (4235 unigenes). Although the eggNOG annotation had the most annotation numbers, the most common annotation was “function unknown” (27,338 unigenes).

**Figure 5:**
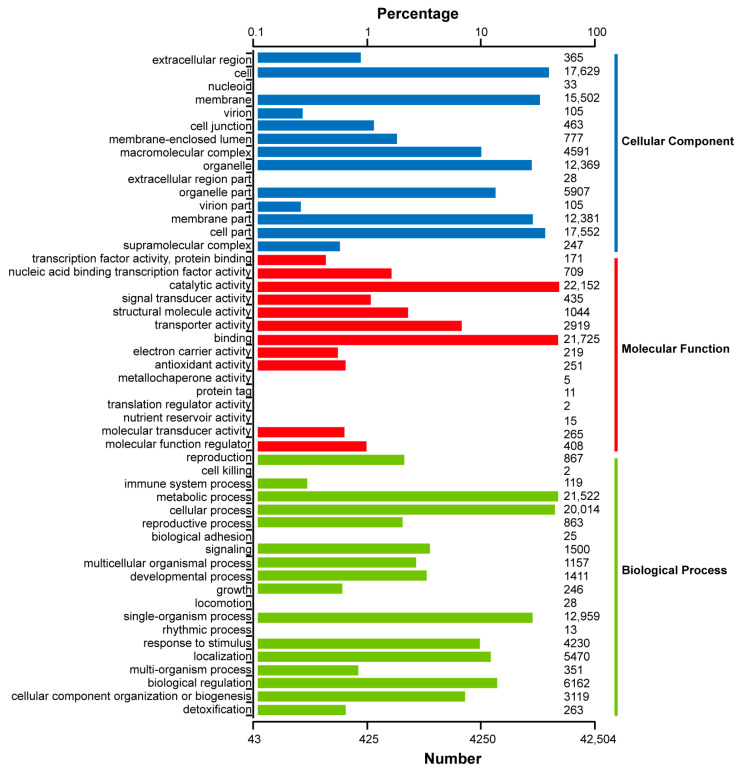
GO functional classification of the consensus sequence.

For the GO analysis, 42,504 unigenes were annotated into three categories—molecular function, cellular component, and biological process (Figure 5; Table S5). Most transcripts clustered into the “catalytic activity” (22,152 unigenes), “binding” (21,725 unigenes), “metabolic process” (21,522 unigenes), and “cellular process” (20,014 unigenes) categories. In addition, the KEGG annotation is also illustrated in Table S4.

### 2.4. Identification of Differentially Expressed Transcripts (DETs)

**Figure 6:**
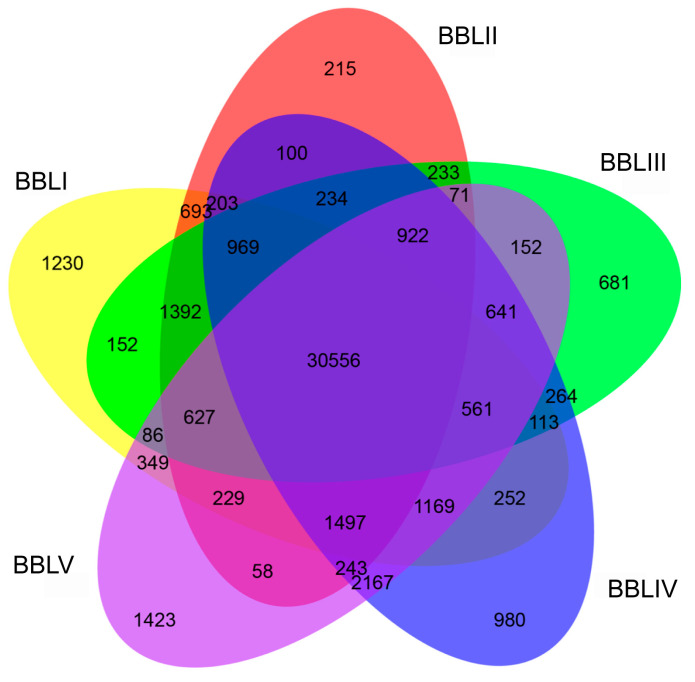
Venn diagram of DETs for five developmental stages.

**Figure 7:**
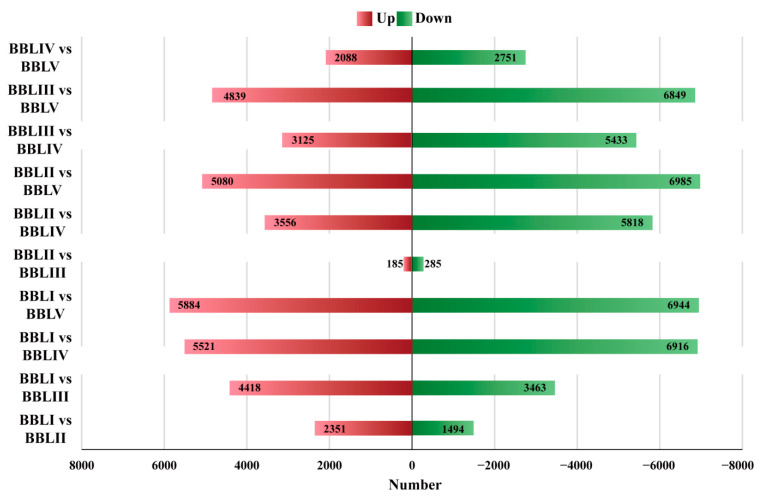
Up-regulated and down-regulated DETs in different comparisons.

Overall, 30,556 transcripts were simultaneously expressed in the leaves of five different stages (Figure 6). Meanwhile, 4529 DETs exhibited stage-specific expression, with 1230, 215, 681, 980, and 1423 transcripts specifically expressed in BBLI, BBLII, BBLIII, BBLIV, and BBLV, respectively. To identify the key genes involved in terpenoid biosynthesis in *B. balsamifera*, we identified 25,200 DETs in 10 combinations (BBLI vs. BBLII, BBLI vs. BBLIII, BBLI vs. BBLIV, BBLI vs. BBLV, BBLII vs. BBLIII, BBLII vs. BBLIV, BBLII vs. BBLV, BBLIII vs. BBLIV, BBLIII vs. BBLV, and BBLIV vs. BBLV) based on NGS sequencing data. Among all of the DETs, the highest number of transcripts (12,828)—comprising 5884 up-regulated genes and 6944 down-regulated genes—were differentially expressed between BBLI and BBLV. This suggested a large biological difference between BBLI and BBLV. However, only a few of the transcripts (470) were differentially expressed between BBLII and BBLIII (Figure 7).

### 2.5. Identification of TFs—1–2000

TFs are a kind of protein that can bind with specific motifs of DNA regulating transcription efficiency, which can affect cell growth, differentiation, and function. In the full-length transcriptome of *B. balsamifera*, 2423 TFs belonging to 67 different TF families were annotated. After removing the TFs with extremely low expression (FPKM < 1), 119 C3Hs, 109 bHLHs, 110 bZIPs, 102 MYB-related TFs, 105 AP2s, and 100 C2H2s were the top six TF families (Table 3). MYB, bHLH, WRKY, and bZIP TFs play a vital role in the regulation of terpenoid biosynthesis in many plant species, such as *Artemisia annua*, *Arabidopsis thaliana*, and *Catharanthus roseus* [24,25,26,27,28,29]. In *B. balsamifera*, most of the bZIPs, bHLHs, and WRKYs had the highest expression level in BBLV. However, nearly half of 88 MYBs showed the highest expression in BBLI. These results reveal that the MYBs play an important role in terpenoid biosynthesis.

### 2.6. Identification of Hub Genes Involved in Terpenoid Biosynthesis

**Figure 8:**
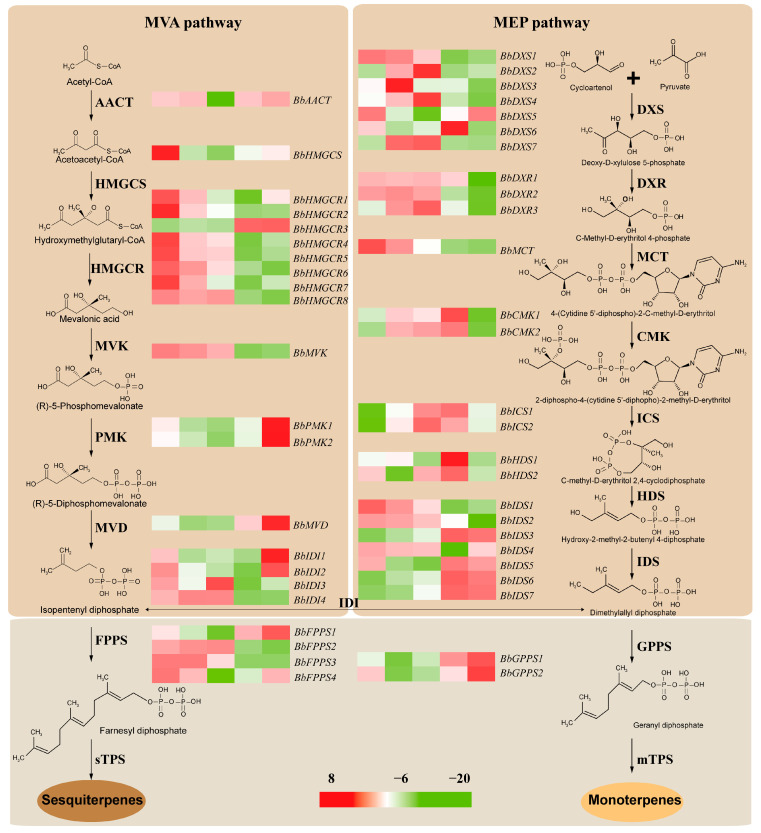
Heatmap of DETs involved in terpenoid backbone biosynthesis.

After removing genes with extremely low expression (FPKM < 1), a total of 116 genes were considered as DETs involved in terpenoid biosynthesis and require extensive investigation in the future. In the MVA pathway, there were 14 genes were identified as DETs related to IPP biosynthesis, including one *BbAACT*, 1 *BbHMGCS*, 8 *BbHMGCRs*, 1 *BbMVK*, *2 BbPMKs*, and 1 *BbMVD*. As the first key gene involved in the MVA pathway, *BbAACT* showed a higher expression level at four developmental stages except for BBLIII, and *BbHMGCS* showed the highest expression in BBLI and the lowest expression in BBLIII. Almost all *BbHMGCRs* and one *BbMVK* had similar expression trends, with the highest expression in BBLI and the lowest expression in BBLIV. However, *BbHMGCR3* had a different expression trend, with the highest expression in BBLV, which was similar to two *BbPMKs* and one *BbMVK* (Figure 8, Table S6). In the MEP pathway, a total of 24 DETs related to DMAPP biosynthesis were identified, comprising 7 *BbDXSs*, 3 *BbDXRs*, 1 *BbMCT*, 2 *BbCMKs*, 2 *BbICSs*, 2 *BbHDSs*, and 7 *BbIDSs*. There was no significant expression trend in the seven *BbDXSs*, but most showed the lowest expression level in BBLIV or BBLV. Three *BbDXRs* had relatively higher expression levels at four developmental stages except for BBLV. *BbMCT* showed a significant downward trend from BBLI to BBLV; however, two *BbCMKs* had an increasing trend from BBLI to BBLIV, with the lowest expression in BBLV. In contrast, two *BbICSs* showed the lowest expression in BBLI and the highest expression in BBLIII or BBLIV. The seven *BbIDSs*, *BbIDS3*, *BbIDS5*, *BbIDS*6, and *BbIDS7* displayed the highest expression in BBLIV and BBLV, whereas *BbIDS1*, *BbIDS2* and *BbIDS4* displayed the lowest expression in BBLIV or BBLV. Meanwhile, four *BbIDIs*, four *BbFPPSs*, and two *GPPSs* were also identified and their expression is illustrated in Figure 8 and Table S6.

**Figure 9:**
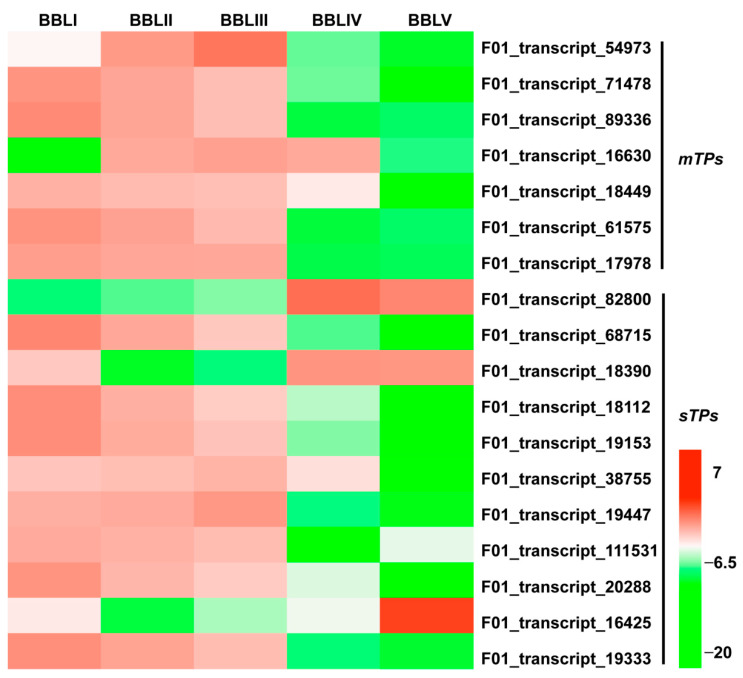
Heatmap of *mTPSs* and *sTPSs* in five developmental stages.

Monoterpenoids and sesquiterpenoids are the main active compounds of the essential oils in *B. balsamifera*. Until now, monoterpenoid synthases (mTPSs) and sesquiterpenoid synthases (sTPSs) in *B. balsamifera* have rarely been reported. In this study, 7 *mTPSs* and 11 *sTPSs* were identified, respectively. Most *mTPSs* and *sTPSs* showed a gradually decreasing trend from BBLI to BBLV and the highest expression in BBLI or BBLII. Only one *mTPS* displayed the lowest expression in BBLI, which was different from the other *mTPSs* (Figure 9, Table S6). However, three *sTPSs* showed completely different expression trends, with the highest expression in BBLIV or BBLV and the lowest expression in BBLII. These genes may play special roles in the biosynthesis of terpenoids in *B. balsamifera*.

### 2.7. RT-qPCR Validation of DETs

**Figure 10:**
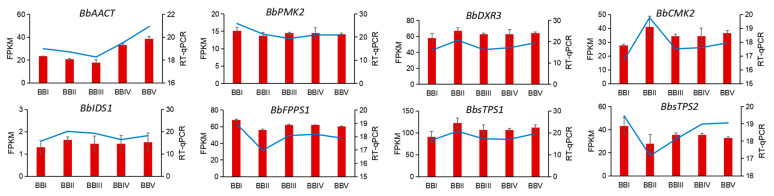
RT-qPCR validation of eight randomly selected genes in *B. balsamifera*. Red bar represents FPKM value, blue line represents RT-PCR results.

Eight DETs were randomly selected to verify the gene expression levels using a real-time qPCR (RT-qPCR) test. The results showed that the relative expression levels of *BbPMK2*, *BbFPPS1*, and *BbsTPS2* were higher in BBLI, and the relative expression levels of *BbDXR3*, *BbCMK2*, *BbIDS1*, and *BbsTPS1* were higher in BBLII. Meanwhile, *BbACCT* showed greater expression in BBLV than in other stages (Figure 10). The RT-qPCR data were in good agreement with the FPKM value in the transcriptome, which proves the accuracy of the transcriptome data.

## 3. Discussion

According to previous reports, the essential oils in *B. balsamifera* leaves are mainly composed of monoterpenoids and sesquiterpenoids, such as (*E*)-caryophyllene, *L*-borneol, longifolene, camphor, *γ*-gurjunene [30,31,32,33]. In this study, the analysis revealed that monoterpenoids and sesquiterpenoids are the main compounds of the essential oils. Among these, the highest levels of monoterpenoids and sesquiterpenoids are *L*-borneol and 2-methylene-4,8,8-trimethyl-4-vinylbicyclo[5.2.0]nonane, respectively. However, the molecular mechanism of terpenoid biosynthesis in *B. balsamifera* is still not clear. To our knowledge, the reference genome of *B. balsamifera* has not yet been published, and transcriptome information for *B. balsamifera* has been rarely reported, which has restricted the development of the molecular biology of *B. balsamifera*. Due to its fast speed, high precision, and low cost, RNA-Seq has become a popular sequencing technology; therefore, RNA-Seq has been used for the transcriptome sequencing of *B. balsamifera* based on the HiSeq 2000 platform, which yielded 100,341 unigene fragments [34]. However, RNA-Seq technology often fails to obtain or assemble complete transcripts. In addition, SMRT technology could provide longer reads of transcripts; however, the accuracy of transcripts generated using SMRT is lower than that using RNA-Seq. In this study, a combination of NGS and full-length transcriptome sequencing was first used to generate high-quality transcriptome data for *B. balsamifera*. This yielded 333,860 CCS reads with a mean read length of 2514 bp, and 65,045 non-redundant transcript isoforms were also obtained. After blasting with eight public databases, 59,958 (92.18%) transcripts were successfully functionally annotated.

TFs play an important role in the transcriptional regulation of monoterpenoids and sesquiterpenoids. For example, bHLH4 and bHLH6 can improve the production of monoterpenoids in butterfly orchids [35], WRKY 1 can promote the biosynthesis of sesquiterpenoids artemisinin in *Artemisia annua* [36], and MsMYB can suppress the expression of geranyl diphosphate synthase [37]. In this study, 2423 TFs distributed in different TF families were identified, including 88 MYBs, 109 bHLHs, 110 bZIPs, and 81 WRKYs. Interestingly, half of the MYBs were mainly expressed in BBLI, which was different from bHLH, bZIP, and WRKY. Therefore, future research should focus on the role of MYBs in the regulation of terpenoid biosynthesis in *B. balsamifera*.

In comparison with the gene expression levels of leaves at different developmental stages, 4529 shared DETs were identified, and further exploration was required to discover their functions. To reveal the structural genes responsible for terpenoid biosynthesis, some candidate transcripts were identified using KEGG pathway annotation. The results revealed that 38 transcripts were assigned to “terpenoid backbone biosynthesis”, among which 14 and 24 transcripts are involved in the MVA and MEP pathways, respectively. AACT is the first enzyme of the MVA pathway and is essential for terpenoid backbone biosynthesis [38]. Only one *BbAACT* was identified, which showed higher expression levels at four developmental stages except for BBLIII in *B. balsamifera*. For the other genes involved in the MVA pathway, almost all decreased gradually during the development of leaves in *B. balsamifera*, including one *BbHMGCs*, seven *BbHMGCRs*, and one *BbMVK*. However, the expression levels of two *BbPMKs* increased gradually in the late development stages, consistent with MiPMK in mango during ripening [39]. One *BbMVD* also displayed a similar expression trend from BBLII to BBLV. In the MEP pathway, all related genes showed no obvious expression trend. It is worth noting that four *BbIDSs* showed higher expression levels in BBLIV or BBLV. Based on previous studies, IDS enzymes function at an important branch point in the biosynthesis of different terpenoids, such as monoterpenoids, sesquiterpenoids, diterpenoids, triterpenoids, and tetraterpenoids [40]. Thus, BbIDSs may play an important role in determining the type of terpenoids.

In addition, as the key enzymes for terpenoid biosynthesis, TPSs can catalyze DMAPP and IPP to form various terpenoids. Recently, more and more TPSs have been identified in medicinal plants. For example, 11 *AaTPSs* were identified in *Angelica archangelica* using RNA-Seq by Suenaga-Hiromori M. et al. [41]; *AaTPSs1-AaTPSs5*, *AaTPSs6-AaTPSs10*, and *AaTPSs11* were responsible for monoterpenoid, sesquiterpenoids, and diterpenoids, respectively. In this study, a total of 18 transcripts were identified as *TPSs*, wherein 11 transcripts were involved in “sesquiterpenoid and triterpenoid biosynthesis” and seven transcripts related to “monoterpenoid biosynthesis”. All these transcripts play important roles in terpenoid biosynthesis in *B. balsamifera* and can potentially be used as target markers for breeding programs aimed at increasing terpenoid production in *B. balsamifera*.

## 4. Materials and Methods

### 4.1. Plant Materials

The fresh leaves from five different developmental stages (named BBLI, BBLII, BBLIII, BBLIV, and BBLV) and the root (BBR) and stem (BBS) of *B. balsamifera* were collected from Red River (Luodian County, Guizhou Province, China). BBLI, BBLII, BBLIII, BBLIV, and BBLV represent leaf buds, small leaves, young leaves, mature leaves, and old leaves, respectively. Each sample was washed with flowing water, surface-dried with sterile filter paper, quick-frozen in liquid nitrogen, and then stored at −80 °C until RNA isolation.

### 4.2. GC–MS Analysis of B. balsamifera Essential Oils

The essential oils from the *B. balsamifera* leaves were extracted using headspace solid-phase microextraction (HS-SPME) after grinding with liquid nitrogen in a mortar, equipped with a 100 μm polydimethylosiloxane (PDMS) fiber (Supelco, Bellefonte, PA, USA) and analyzed using a 7890B-7000D gas chromatograph (Agilent Technologies, Santa Clara, CA, USA). The instrument was equipped with an Agilent DB-5MS capillary column (30 m × 0.25 mm × 0.25 μm film) and helium was used as the carrier gas at a flow rate of 1.0 mL/min. The GC oven was maintained at 40 °C for 5 min, gradually increased to 280 °C at a rate of 6 °C/min, and then held for 5 min. The original data obtained were first extracted using Mass Hunter software (Agilent) to obtain the mass-to-charge ratio, retention time, peak area, and other information for the characteristic peak. Then, the quality and quantity of the metabolites were analyzed based on the MWGC database.

### 4.3. RNA Extraction and Sequencing

A total of 21 RNA samples (each in triplicate) were extracted using Trizol (Sangon Biotech, Shanghai, China). The quality and quantity of the extracted RNA were determined using agarose gel electrophoresis and the Nanodrop 2000 system. For NGS sequencing, mRNA was enriched with magnetic beads from the total RNA of BBLI, BBLII, BBLIII, BBLIV, and BBLV, then the cDNAs were synthesized using random hexamers and purified using AMPure XP beads. Fifteen cDNA libraries were constructed using PCR enrichment. The concentration and quality of these cDNA fragments were measured using an Agilent 2100 bioanalyzer. After passing the quality inspection, 15 libraries were sequenced using the Illumina platform (Biomarker Technologies, Beijing, China). In addition, the library used for the full-length transcriptome sequencing was composed using equal quantities of total RNA from all 21 individual samples. The full-length transcriptome sequencing was completed using the PacBio platform (Biomarker Technologies, Beijing, China) based on SMRT technology.

### 4.4. Full-Length Transcriptome Analysis

For full-length transcriptome analysis, raw reads were processed into circular consensus (CCS) reads according to having full passes ≥ 3 and an accuracy of sequence > 90%. The full-length non-chimeric reads in the CCS were screened and clustered into consensus isoforms. Then, non-redundant isoforms were generated using CD-HIT software and the integrity was assessed using BUSCO software (version 5.4.7, https://busco.ezlab.org/, accessed on 2 February 2020) [42]. Subsequently, the coding sequence (CDS), SSR types, and transcription factors (TFs) were also analyzed using TransDecoder (version 5.5.0, https://github.com/TransDecoder/, accessed on 3 February 2020), MISA (version 2.1, https://webblast.ipk-gatersleben.de/misa/index.php?action=1, accessed on 3 February 2020), and iTAK (version 1.6, http://itak.feilab.net/cgi-bin/itak/index.cgi, accessed on 3 February 2020) [43], respectively. Meanwhile, the lncRNAs were also predicted using the Coding Potential Calculator (CPC2, version 2.0 β, http://cpc2.gao-lab.org/, accessed on 3 February 2020) [44], the Coding-Non-Coding Index (CNCI, https://github.com/www-bioinfo-org/CNCI#install-cnci, accessed on 5 February 2020) [45], Protein family (Pfam, http://pfam.xfam.org/, accessed on 5 February 2020) [46], and the Coding Potential Assessment Tool (CPAT, http://lilab.research.bcm.edu/cpat/, accessed on 5 February 2020) [47].

### 4.5. Functional Annotation of Non-Redundant Isoforms

The functional annotation of non-redundant isoforms was performed by blasting with various databases, such as the Non-Redundant (NR) Protein Sequence Database [48], Swissprot, Gene Ontology (GO) [49], Clusters of Orthologous Groups (COG) [50], euKaryotic Ortholog Groups (KOG) [51], Pfam, and the Kyoto Encyclopedia of Genes and Genomes (KEGG) [52].

### 4.6. Identification of Differentially Expressed Transcripts (DETs)

For the NGS analysis, clean data were obtained from raw data after a series of quality-control procedures, such as primer deletion and lowest-quality-read filtering. Gene expression levels were identified by calculating the total numbers of the fragments mapping to each transcript and normalization using fragments per kilobase of transcript per million mapped reads (FPKM). Differential expression analysis of two groups was performed using DESeq2. The resulting *p*-values were adjusted using Benjamini and Hochberg’s approach for controlling the false discovery rate; genes with an FDR < 0.01 and a fold change ≥ 2 were assigned as DETs.

### 4.7. Real Time-Quantitative PCR (RT-qPCR) Validation

Eight random genes were selected for RT-qPCR analysis to confirm the RNA-Seq results. The total RNA of the leaves at five different developmental stages was isolated with Trizol, and from each sample, ~0.5 µg of RNA was used to synthesize the first-strand cDNAs. The specific primers used are listed in Table S7. Each PCR reaction comprised of template cDNA (1 µL), forward primer and reverse primer (0.8 µL), 2× *TransStart*^®^ Green qPCR SuperMix (10 µL), and nuclease-free water (9.2 µL). The PCR amplification was carried out as follows: 60 s at 95 °C followed by 40 cycles of 5 s at 95 °C and 60 s at 60 °C. After normalization with the *actin* gene as an internal reference, gene expression levels were calculated using the 2^−∆∆Ct^ method. Three replicates were conducted for each sample.

## 5. Conclusions

In this study, transcriptomic and metabolomic data for *B. balsamifera* were first obtained using a combination of NGS and full-length transcriptome sequencing and GC–MS. Through structural analysis and functional annotation, a large amount of accurate and complete transcripts was identified, including DETs and TFs at five different developmental stages in *B. balsamifera*. These results reveal that the regulatory mechanism of terpenoid biosynthesis is complicated and needs further research. These data provide a valuable basis for gene discovery, molecular breeding, and the metabolic engineering of *B. balsamifera*.

## Figures and Tables

**Table 1:** Summary of metabolomics analysis using GC–MS.

Number	Name of Compound	Formula	Retention Index	Count Per Second
1	*L*(-)-Borneol	C_10_H_18_O	1.17 × 10^3^	5186.23
2	(-)-Terpinen-4-ol	C_10_H_18_O	1.18 × 10^3^	86.64
3	Linalool	C_10_H_18_O	1.10 × 10^3^	23.27
4	(-)-Orthodene	C_10_H_16_	1.04 × 10^3^	14.13
5	Camphene	C_10_H_16_	9.49 × 10^2^	12.40
6	(-)-Bornyl acetate	C_12_H_20_O_2_	1.29 × 10^3^	8.60
7	(+)-2-Bornanone	C_10_H_16_O	1.15 × 10^3^	6.84
8	1,3-Cyclohexadiene	C_10_H_16_	1.04 × 10^3^	5.86
9	*L*-*α*-Terpineol	C_10_H_18_O	1.20 × 10^3^	1.80
10	*p*-Mentha-1,5,8-triene	C_10_H_14_	1.13 × 10^3^	1.69
11	Pinocarvone	C_10_H_14_O	1.16 × 10^3^	0.54
12	1-(2-Ethyl-3-cyclohexenyl)ethanol	C_10_H_18_O	1.14 × 10^3^	0.19
13	Allo-ocimene	C_10_H_16_	1.14 × 10^3^	0.19
14	*α*-Thujene	C_10_H_16_	9.25 × 10^2^	0.19
15	*cis*-Sabinenhydrate	C_10_H_1_8O	9.77 × 10^2^	0.07
16	2-Bornene	C_10_H_16_	9.73 × 10^2^	0.05
17	2-methylene-4,8,8-trimethyl-4-vinylbicyclo[5.2.0]nonane	C_15_H_24_	1.43 × 10^3^	1272.53
18	1R,4R,7R,11R-1,3,4,7-Tetramethyltricyclo[5.3.1.0(4,11)]undec-2-ene	C_15_H_24_	1.35 × 10^3^	942.58
19	*β*-Copaene	C_15_H_24_	1.43 × 10^3^	661.04
20	(-)-*α*-Gurjunene	C_15_H_24_	1.44 × 10^3^	231.35
21	(+)-*epi*-Bicyclosesquiphellandrene	C_15_H_24_	1.49 × 10^3^	56.64
22	*β*-Maaliene	C_15_H_24_	1.41 × 10^3^	29.59
23	*γ*-Cadinene	C_15_H_24_	1.52 × 10^3^	14.22
24	*α*-Calacorene	C_15_H_20_	1.55 × 10^3^	8.52
25	Cadina-3,5-diene	C_15_H_24_	1.38 × 10^3^	8.17
26	Epizonarene	C_15_H_24_	1.51 × 10^3^	7.33
27	*γ*-Selinene	C_15_H_24_	1.50 × 10^3^	6.82
28	*β*-Cadinene	C_15_H_24_	1.49 × 10^3^	5.62
29	Rosifoliol	C_15_H_26_O	1.61 × 10^3^	5.04
30	*β*-Elemen	C_15_H_24_	1.39 × 10^3^	4.64
31	*γ*-Muurolene	C_15_H_24_	1.37 × 10^3^	2.48
32	(-)-Clovene	C_15_H_24_	1.37 × 10^3^	2.00
33	*α*-Corocalene	C_15_H_20_	1.62 × 10^3^	1.24
34	6-Isopropyl-1,4-dimethylnaphthalene	C_15_H_18_	1.78 × 10^3^	0.53
35	*β*-Guaiene	C_15_H_24_	1.56 × 10^3^	0.36
36	Kessane	C_15_H_26_O	1.54 × 10^3^	0.26

**Table 2:** Summary of PacBio single-molecule real-time (SMRT) sequencing.

cDNA Size	Number of Unpolished Consensus Isoforms	Mean Unpolished Consensus Isoform Read Length	Number of Polished HQ Isoforms	Percent of Polished HQ Isoforms (%)
1–6 K	116,656	2244	116,639	99.99
All	116,656	2244	116,639	99.99

**Table 3:** Top 21 TFs in *B. balsamifera*.

Order Number	Name of TF Family	Number of Transcripts	Number of TFs with Highest Expression (FPKM > 1)				
			BBLI	BBLII	BBLIII	BBLIV	BBLV
1	C3H	119	34	2	3	16	64
2	bZIP	110	20	9	3	23	55
3	bHLH	109	24	14	14	24	33
4	AP2	105	28	6	10	21	40
5	MYB-related	102	18	2	13	27	42
6	C2H2	100	22	6	3	24	45
7	NAC	94	10	3	2	13	66
8	MYB	88	39	4	7	16	22
9	GRAS	84	16	4	5	9	50
10	WRKY	81	11	1	1	19	49
11	HB-HD-ZIP	69	34	1	0	8	26
12	B3-ARF	68	48	4	1	12	3
13	Trihelix	68	24	5	3	11	25
14	RWP-RK	56	11	0	5	22	18
15	GARP-G2-like	49	9	0	7	11	22
16	HSF	46	10	0	1	20	15
17	SBP	37	17	2	1	2	15
18	TCP	37	9	7	3	10	8
19	TUB	32	5	2	2	11	12
20	C2C2-Dof	32	5	5	4	7	11
21	C2C2-GATA	31	16	3	5	2	5

## Data Availability

All data are contained within the article and the Supplementary Materials.

## Supplementary Materials

The following supporting information can be downloaded at: https://www.mdpi.com/article/10.3390/genes15030285/s1. Figure S1: The integrity of transcriptome. Table S1: Overview of assembly and quality evaluation of the *B. balsamifera* RNA-Seq clean data. Table S2: Annotation of transcripts against eight different public databases. Table S3: KOG pathway annotation for *B. balsamifera*. Table S4: GO annotation of the *B. balsamifera* transcripts. Table S5: KEGG pathway annotation for *B. balsamifera*. Table S6: Differentially expressed genes involved in mono- and sesquiterpenoid biosynthesis. Table S7: Primers used in the validation experiment of gene expression using RT-qPCR.
