# Supplementary material for: Full-Length Transcriptome Sequencing and RNA-Seq Analysis Offer Insights into Terpenoid Biosynthesis in Blumea balsamifera (L.) DC
Source: Genes (Basel). 2024 Feb 24;15(3):285. doi: 10.3390/genes15030285 (PMC10970515; doi:10.3390/genes15030285)
Supplement: Supplementary file 1 [file genes-15-00285-s001.zip › genes-2856663-SI.pdf]

Full-Length Transcriptome Sequencing and RNA-Seq Analysis Offer Insights into Terpenoid Biosynthesis in *Blumea balsamifera* (L.) DC.

Zhigang Ju [1,†], Lin Liang [1,†], Yaqiang Zheng [1], Hongxi Shi [1], Wenxuan Zhao [1], Wei Sun [2,*], Yuxin Pang [1,3,*]

1 Phamarcy College, Guizhou University of Traditional Chinese Medicine, Guiyang, China; juzhigang088@gzy.edu.cn (Z.J.); liangl187@163.com (L.L.); zhengyaqiang131@gzy.edu.cn (Y.Z.); shihongxi055@gzy.edu.cn (H.S.); zhaowenxuan0602@163.com(W.Z.)
2 Key Laboratory of State Forestry Administration on Biodiversity Conservation in Karst Mountain Area of Southwest of China, School of Life Science, Guizhou Normal University, Guiyang, China
3 Yunfu Branch, Guangdong Laboratory for Lingnan Modern Agriculture, Yunfu, China
* Correspondence: sunwei@gznu.edu.cn (W.S.); pyxmarx@gzy.edu.cn (Y.P.)
† These authors contributed equally to this work.

1 Supplementary Figures

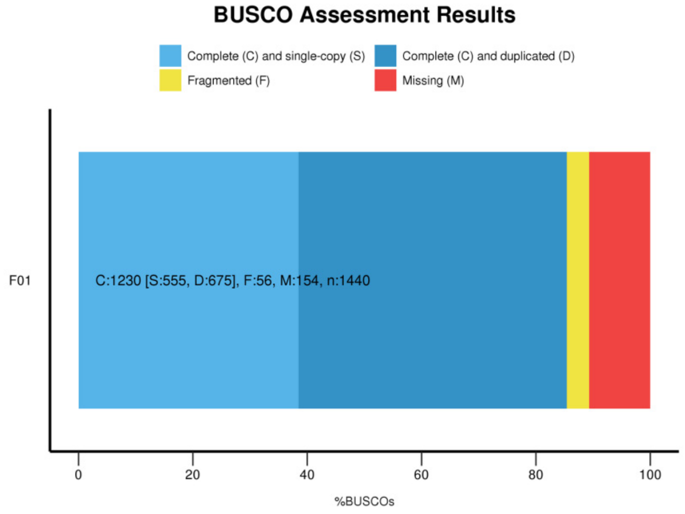

Figure S1. The integrity of transcriptome.

2 Supplementary Tables

Table S1. Overview of assembly and quality evaluation of the *B.balsamifera* RNA-seq clean data.

Sample ID	Obtained	Obtained Base (bp)	GC(%)	Q20(%)	Q30(%)
BBLI1	22304507	6672183832	45.42	98.59	95.54
BBLI2	30028801	8971709662	45.85	98.59	95.56
BBLI3	26587290	7940250548	45.3	98.57	95.52
BBLII1	23811488	7119260638	45.82	98.64	95.65
BBLII2	28310526	8468387880	45.8	98.63	95.66
BBLII3	29268192	8748374770	46.01	98.62	95.66
BBLIII1	19753373	5913498110	46.06	98.62	95.73
BBLIII2	22334771	6677646544	45.59	98.68	95.76
BBLIII3	26665691	7982867416	46.09	97.98	94.59
BBLIV1	32328414	9668285068	43.73	98.45	95.18
BBLIV2	29922419	8950779888	43.9	98.54	95.4
BBLIV3	20796053	6211788576	43.74	98.52	95.33
BBLV1	22955120	6854539798	43.74	98.56	95.47
BBLV2	22001365	6578150698	43.82	98.64	95.68
BBLV3	24929085	7456014906	43.64	98.57	95.43
Average	25466473	7614249222	44.97	98.55	95.48

Table S2. Annotation of transcripts against eight different public databases.

Annotated	Number of	Annotated	300<=length	length>=1000
COG	25,569	39.31	1,193	24,374
GO	42,504	65.35	2,815	39,681
KEGG	27,306	41.98	1,807	25,494
KOG	39,064	60.06	2,181	36,873
Pfam	49,022	75.37	2,759	46,263
SwissProt	43,396	66.72	2,515	40,869
eggNOG	57,793	88.85	3,654	54,125
Nr	59,703	91.79	3,931	55,751
All	59,957	92.18	3,952	55,982

Table S3. KOG pathway annotation of *B.balsamifera*.

#ID	Class Name	Numbers
R	General function prediction only	8455
T	Signal transduction mechanisms	4268
O	Posttranslational modification, protein turnover, chaperones	4235
K	Transcription	2421
G	Carbohydrate transport and metabolism	2377
S	Function unknown	2287
A	RNA processing and modification	2186
U	Intracellular trafficking, secretion, and vesicular transport	2164
J	Translation, ribosomal structure and biogenesis	1976
E	Amino acid transport and metabolism	1737
I	Lipid transport and metabolism	1586
C	Energy production and conversion	1426
Q	Secondary metabolites biosynthesis, transport and catabolism	1405
L	Replication, recombination and repair	1268
P	Inorganic ion transport and metabolism	1240
D	Cell cycle control, cell division, chromosome partitioning	926
Z	Cytoskeleton	843
M	Cell wall/membrane/envelope biogenesis	582
B	Chromatin structure and dynamics	577
H	Coenzyme transport and metabolism	468
F	Nucleotide transport and metabolism	465
V	Defense mechanisms	266

Y	Nuclear structure	228
W	Extracellular structures	114
N	Cell motility	20

Table S4. KEGG pathway annotation of *B.balsamifera*.

Pathway_level1	Pathway_level2	Number_of_Genes
Cellular Processes	Transport and catabolism	1308
Environmental Information Processing	Membrane transport	133
Environmental Information Processing	Signal transduction	786
Genetic Information Processing	Folding, sorting and degradation	2024
Genetic Information Processing	Replication and repair	1004
Genetic Information Processing	Transcription	999
Genetic Information Processing	Translation	2455
Metabolism	Amino acid metabolism	2069
Metabolism	Biosynthesis of other secondary metabolites	519
Metabolism	Carbohydrate metabolism	3700
Metabolism	Energy metabolism	1051
Metabolism	Global and overview maps	2389
Metabolism	Glycan biosynthesis and metabolism	812
Metabolism	Lipid metabolism	1415
Metabolism	Metabolism of cofactors and vitamins	870
Metabolism	Metabolism of other amino acids	616
Metabolism	Metabolism of terpenoids and polyketides	441
Metabolism	Nucleotide metabolism	852
Organismal Systems	Environmental adaptation	682

Table S5. GO annotation of the *B.balsamifera* transcripts.

Functional Attributes	Term Name	Number
cellular component	cell	17629
cellular component	cell part	17552
cellular component	membrane	15502

cellular component	membrane part	12381
cellular component	organelle	12369
cellular component	organelle part	5907
cellular component	macromolecular complex	4591
cellular component	membrane-enclosed lumen	777
cellular component	cell junction	463
cellular component	extracellular region	365
cellular component	supramolecular complex	247
cellular component	virion	105
cellular component	virion part	105
cellular component	nucleoid	33
cellular component	extracellular region part	28
molecular function	catalytic activity	22152
molecular function	binding	21725
molecular function	transporter activity	2919
molecular function	structural molecule activity	1044
molecular function	nucleic acid binding transcription factor activity	709
molecular function	signal transducer activity	435
molecular function	molecular function regulator	408
molecular function	molecular transducer activity	265
molecular function	antioxidant activity	251
molecular function	electron carrier activity	219
molecular function	transcription factor activity, protein binding	171
molecular function	nutrient reservoir activity	15
molecular function	protein tag	11
molecular function	metallochaperone activity	5
molecular function	translation regulator activity	2
biological process	metabolic process	21522
biological process	cellular process	20014
biological process	single-organism process	12959
biological process	biological regulation	6162

biological process	localization	5470
biological process	response to stimulus	4230
biological process	cellular component organization or biogenesis	3119
biological process	signaling	1500
biological process	developmental process	1411
biological process	multicellular organismal process	1157
biological process	reproduction	867
biological process	reproductive process	863
biological process	multi-organism process	351
biological process	detoxification	263
biological process	growth	246
biological process	immune system process	119
biological process	locomotion	28
biological process	biological adhesion	25
biological process	rhythmic process	13
biological process	cell killing	2

Table S6. Differentially expressed genes involved in mono- and sesquiterpenoids biosynthesis.

		Gene ID	Gene Name	BBI	BBII	BBIII	BBIV	BBV
**MVA**	*AACT*	F01_transcript_65843	*BbAACT*	0.33	0.54	0.00	0.40	1.43
	HMGCS	F01_transcript_21012	*BbHMGCS*	68.28	32.48	50.38	50.38	44.41
	HMGCR	F01_transcript_38369	*BbHMGCR1*	4.01	2.68	3.34	3.34	3.12
		F01_transcript_48506	*BbHMGCR2*	10.71	3.01	6.86	6.86	5.58
		F01_transcript_15357	*BbHMGCR3*	24.74	35.56	30.15	30.15	31.96
		F01_transcript_66586	*BbHMGCR4*	18.00	5.70	11.85	11.85	9.80
		F01_transcript_64189	*BbHMGCR5*	35.27	10.73	23.00	23.00	18.91
		F01_transcript_11551	*BbHMGCR6*	2.33	2.39	2.36	2.36	2.37
		F01_transcript_87819	*BbHMGCR7*	59.27	20.21	39.74	39.74	33.23
		F01_transcript_71737	*BbHMGCR8*	2.60	2.20	2.40	2.40	2.33
	MVK	F01_transcript_61575	*BbMVK*	240.77	126.96	183.86	183.86	164.89

	PMK	F01_transcript_87808	*BbPMK1*	2.59	1.52	2.06	2.06	1.88
		F01_transcript_107526	*BbPMK2*	5.18	3.71	4.45	4.45	4.20
	MVD	F01_transcript_66651	*BbMVD*	27.14	21.53	22.31	31.50	45.29
	DXS	F01_transcript_111516	*BbDXS1*	30.45	25.71	28.08	28.08	27.29
		F01_transcript_9408	*BbDXS2*	8.68	21.87	15.27	15.27	17.47
		F01_transcript_105023	*BbDXS3*	27.19	43.65	35.42	35.42	38.16
		F01_transcript_79292	*BbDXS4*	28.40	32.14	30.27	30.27	30.89
		F01_transcript_9387	*BbDXS5*	155.90	20.67	88.29	88.29	65.75
		F01_transcript_8481	*BbDXS6*	1.63	1.34	1.49	1.49	1.44
		F01_transcript_10126	*BbDXS7*	14.17	29.34	21.75	21.75	24.28
	DXR	F01_transcript_116620	*BbDXR1*	4.90	3.71	4.31	4.31	4.11
		F01_transcript_20224	*BbDXR2*	30.92	34.46	32.69	32.69	33.28
		F01_transcript_78679	*BbDXR3*	57.99	67.20	62.60	62.60	64.13
**MEP**	*MCT*	F01_transcript_115715	*BbMCT*	17.58	16.06	13.99	11.90	11.57
	CMK	F01_transcript_54575	*BbCMK1*	12.82	16.48	14.65	14.65	15.26
		F01_transcript_107216	*BbCMK2*	27.79	40.99	34.39	34.39	36.59
	ICS	F01_transcript_31660	*BbICS1*	77.73	144.91	111.32	111.32	122.52
		F01_transcript_34713	*BbICS2*	9.61	17.95	25.04	21.28	15.83
	HDS	F01_transcript_8398	*BbHDS1*	11.24	17.64	14.44	14.44	15.51
		F01_transcript_35639	*BbHDS2*	1.65	0.70	1.18	1.18	1.02
	IDS	F01_transcript_93746	*BbIDS1*	178.36	122.70	150.53	150.53	141.26
		F01_transcript_74597	*BbIDS2*	12.98	11.91	12.45	12.45	12.27
		F01_transcript_50236	*BbIDS3*	30.55	54.24	42.40	42.40	46.34
		F01_transcript_70382	*BbIDS4*	5.16	2.26	3.71	3.71	3.23
		F01_transcript_41275	*BbIDS5*	4.04	1.36	2.70	2.70	2.25
		F01_transcript_47645	*BbIDS6*	24.97	51.04	38.01	38.01	42.35
		F01_transcript_93798	*BbIDS7*	1.30	1.64	1.47	1.47	1.53
	IDI	F01_transcript_90540	*BbIDI1*	2.03	1.11	1.27	1.05	3.84
		F01_transcript_56635	*BbIDI2*	50.91	35.40	29.56	22.29	61.34
		F01_transcript_86158	*BbIDI3*	0.95	0.68	1.24	0.40	0.58
		F01_transcript_30087	*BbIDI4*	77.87	104.03	103.41	17.75	18.70

	F01_transcript_58966	*BbFPPS1*	68.18	56.27	62.23	62.23	60.24
FPPS	F01_transcript_27735	*BbFPPS2*	22.67	28.28	25.47	25.47	26.41
	F01_transcript_115460	*BbFPPS3*	22.73	22.24	22.48	22.48	22.40
	F01_transcript_25698	*BbFPPS4*	68.18	56.27	62.23	62.23	60.24
GPPS	F01_transcript_25607	*BbGPPS1*	37.34	19.91	28.63	28.63	25.72
	F01_transcript_48890	*BbGPPS2*	1.99	0.74	1.37	1.37	1.16
	F01_transcript_79992	*BbGGPPS1*	324.93	732.00	528.46	528.46	596.31
	F01_transcript_50264	*BbGGPPS2*	21.54	18.61	20.07	20.07	19.58
	F01_transcript_44623	*BbGGPPS3*	24.26	39.16	31.71	31.71	34.19
	F01_transcript_26327	*BbGGPPS4*	22.09	38.67	30.38	30.38	33.15
GGPPS	F01_transcript_26324	*BbGGPPS5*	45.64	36.01	40.82	40.82	39.22
	F01_transcript_114832	*BbGGPPS6*	9.35	7.08	8.22	8.22	7.84
	F01_transcript_26324	*BbGGPPS7*	45.64	36.01	40.82	40.82	39.22
	F01_transcript_26327	*BbGGPPS8*	22.09	38.67	30.38	30.38	33.15
	F01_transcript_50264	*BbGGPPS9*	21.54	18.61	20.07	20.07	19.58
	F01_transcript_44623	*BbGGPPS10*	24.26	39.16	31.71	31.71	34.19
	F01_transcript_22680	*BbmTPS1*	17.98	14.79	16.39	16.39	15.85
	F01_transcript_21029	*BbmTPS2*	5.83	7.99	6.91	6.91	7.27
	F01_transcript_21584	*BbmTPS3*	0.08	0.19	0.14	0.14	0.15
	F01_transcript_61177	*BbmTPS4*	3.41	1.91	2.66	2.66	2.41
	F01_transcript_79565	*BbmTPS5*	1.94	3.15	2.54	2.54	2.74
	F01_transcript_21231	*BbmTPS6*	49.81	21.89	35.85	35.85	31.20
	F01_transcript_88949	*BbmTPS7*	8.30	10.65	9.47	9.47	9.86
mTPS	F01_transcript_104125	*BbmTPS8*	6.80	4.64	5.72	5.72	5.36
	F01_transcript_19156	*BbmTPS9*	10.96	9.85	10.41	10.41	10.22
	F01_transcript_52773	*BbmTPS10*	3.04	1.10	2.07	2.07	1.75
	F01_transcript_69023	*BbmTPS11*	16.28	14.24	15.26	15.26	14.92
	F01_transcript_41199	*BbmTPS12*	1.61	4.90	3.26	3.26	3.80
	F01_transcript_71784	*BbmTPS13*	4.70	4.35	4.53	4.53	4.47
	F01_transcript_102473	*BbmTPS14*	0.64	19.08	9.86	9.86	12.93
	F01_transcript_16630	*BbmTPS15*	0.68	27.99	14.34	14.34	18.89

	F01_transcript_58349	*BbmTPS16*	2.15	0.33	1.24	1.24	0.94
	F01_transcript_80988	*BbmTPS17*	1.06	2.72	1.89	1.89	2.16
	F01_transcript_61575	*BbmTPS18*	240.77	126.96	183.86	183.86	164.89
	F01_transcript_89336	*BbmTPS19*	11.77	9.12	10.45	10.45	10.00
	F01_transcript_17978	*BbmTPS20*	58.09	50.56	54.33	54.33	53.07
	F01_transcript_97908	*BbmTPS21*	4.84	9.43	7.13	7.13	7.90
	F01_transcript_29671	*BbmTPS22*	39.72	53.07	46.40	46.40	48.62
	F01_transcript_15530	*BbsTPS1*	0.09	0.04	0.07	0.07	0.06
	F01_transcript_68715	*BbsTPS2*	0.11	0.18	0.15	0.15	0.16
	F01_transcript_18390	*BbsTPS3*	8.71	0.11	4.41	4.41	2.98
	F01_transcript_56882	*BbsTPS4*	0.09	0.00	0.05	0.05	0.03
	F01_transcript_116267	*BbsTPS5*	4.42	8.29	6.35	6.35	7.00
	F01_transcript_18112	*BbsTPS6*	42.79	25.96	34.38	34.38	31.57
	F01_transcript_79063	*BbsTPS7*	2.13	1.30	1.72	1.72	1.58
	F01_transcript_43718	*BbsTPS8*	1.84	0.76	1.30	1.30	1.12
	F01_transcript_102954	*BbsTPS9*	1.18	0.01	0.60	0.60	0.40
	F01_transcript_71478	*BbsTPS10*	30.13	19.76	24.95	24.95	23.22
	F01_transcript_59437	*BbsTPS11*	10.53	7.33	8.93	8.93	8.40
sTPS	F01_transcript_70940	*BbsTPS12*	1.22	0.00	0.61	0.61	0.41
	F01_transcript_54973	*BbsTPS13*	0.84	3.50	2.17	2.17	2.61
	F01_transcript_19153	*BbsTPS14*	17.58	9.77	13.67	13.67	12.37
	F01_transcript_57670	*BbsTPS15*	2.91	1.39	2.15	2.15	1.90
	F01_transcript_17879	*BbsTPS16*	2.41	3.19	2.80	2.80	2.93
	F01_transcript_107626	*BbsTPS17*	22.24	19.03	20.64	20.64	20.10
	F01_transcript_20223	*BbsTPS18*	2.83	3.28	3.06	3.06	3.13
	F01_transcript_78917	*BbsTPS19*	23.51	14.12	18.82	18.82	17.25
	F01_transcript_38755	*BbsTPS20*	0.64	0.87	0.76	0.76	0.79
	F01_transcript_105863	*BbsTPS21*	2.64	1.03	1.84	1.84	1.57
	F01_transcript_17767	*BbsTPS22*	5.88	0.02	2.95	2.95	1.98
	F01_transcript_19447	*BbsTPS23*	6.15	6.60	6.38	6.38	6.45
	F01_transcript_73544	*BbsTPS24*	9.37	3.90	6.64	6.64	5.72

F01_transcript_36829	*BbsTPS25*	43.00	27.84	35.42	35.42	32.89
F01_transcript_28634	*BbsTPS26*	9.52	14.36	11.94	11.94	12.75
F01_transcript_21737	*BbsTPS27*	11.27	8.44	9.86	9.86	9.39
F01_transcript_96118	*BbsTPS28*	3.17	2.07	2.62	2.62	2.44
F01_transcript_111531	*BbsTPS29*	132.81	77.60	105.21	105.21	96.00
F01_transcript_20288	*BbsTPS30*	27.90	14.11	21.01	21.01	18.71
F01_transcript_81074	*BbsTPS31*	5.65	7.22	6.44	6.44	6.70
F01_transcript_16425	*BbsTPS32*	2.29	0.08	1.19	1.19	0.82
F01_transcript_91019	*BbsTPS33*	5.24	3.07	4.16	4.16	3.79
F01_transcript_19333	*BbsTPS34*	106.31	62.55	84.43	84.43	77.14
F01_transcript_73466	*BbsTPS35*	7.90	2.91	5.41	5.41	4.58
F01_transcript_16048	*BbsTPS36*	1.58	2.59	2.09	2.09	2.26

Table S7. Primers used in validation experiment of gene expression by RT-qPCR.

	Gene id	Forward primer	Reverse primer
Bbactin		CGATCATTGATGGCTGG	GCCGAGCGAGAAATTGT
BbAACT	F01_transcript_25951	AAGTATAAGCGATGGTGCGG	GCAGTGGTAAACAATTCAGGG
BbPMK	F01_transcript_107526	CAATCCTAGGTTGCAATGACTTC	GATTCTGGGACTAAAGGGAGC
BbDXR	F01_transcript_78679	AATCACTTTCTCCGACACCTG	CTCTTAACAGAAAACCCACCTTG
BbCMK	F01_transcript_107216	GGAAGGGAAAGAGAATGGGTG	TTCCTTAACCTCGTGCATCC
BbIDS	F01_transcript_93746	CCAGAAGCGATAATTACAACCG	AGCTTCACAGTCACATTCCC
BbFPPS	F01_transcript_58966	AGTGCTCGTGGTTAGTTGTG	TCGAAGACACCCTGAAGATTG
BbsTPS1	F01_transcript_16863	TGACCAGCACGAAGATGAAG	CGTAGAAATGTTGAAAGCTCGC
BbsTPS2	F01_transcript_36829	CCCAAACAATCTGCCATATGC	CATGAGAATACTGTGGCTGAAAG
